# Incidence of infants born small- and large-for-gestational-age in an Italian cohort over a 20-year period and associated risk factors

**DOI:** 10.1186/s13052-016-0254-7

**Published:** 2016-04-26

**Authors:** Valentina Chiavaroli, Valeria Castorani, Paola Guidone, José G. B. Derraik, Marco Liberati, Francesco Chiarelli, Angelika Mohn

**Affiliations:** Department of Paediatrics, University of Chieti, Via dei Vestini 5, 66100 Chieti, Italy; Center of Excellence on Aging, “G. d’Annunzio” University Foundation, University of Chieti, Chieti, Italy; Liggins Institute, University of Auckland, Auckland, New Zealand; Department of Obstetrics and Gynaecology, University of Chieti, Chieti, Italy

**Keywords:** SGA, LGA, Incidence, Gestational diabetes, Obesity, Birth weight, Birth length, Smoking

## Abstract

**Background:**

We assessed the incidence of infants born small-for-gestational-age (SGA) and large-for-gestational-age (LGA) in an Italian cohort over 20 years (1993–2013). Furthermore, we investigated maternal factors associated with SGA and LGA births.

**Methods:**

A retrospective review of obstetric records was performed on infants born in Chieti (Italy) covering every 5^th^ year over a 20-year period, specifically examining data for 1993, 1998, 2003, 2008, and 2013. Infants with birthweight <10^th^ percentile were defined as SGA, and those with birthweight >90^th^ percentile as LGA. Data collected included newborn anthropometry, birth (multiple vs singleton), maternal anthropometry, previous miscarriage, gestational diabetes, hypertension, and smoking during pregnancy.

**Results:**

There were a pooled total of 5896 live births recorded across the 5 selected years. The number of SGA (+60.6 %) and LGA (+90.2 %) births increased considerably between 1993 and 2013. However, there were no marked changes in the incidence of SGA or LGA births (8.3 % and 10.8 % in 1993 versus 7.6 % and 11.7 % in 2013, respectively). Maternal factors associated with increased risk of SGA infants included hypertension, smoking, and previous miscarriage (all *p* < 0.05), while greater pre-pregnancy BMI and gestational diabetes were risk factors for LGA births (all *p* < 0.05).

**Conclusions:**

There was an increase in the number of SGA and LGA births in Chieti over the last two decades, but there was little change in incidence over time. Most maternal factors associated with increased odds of SGA and LGA births were modifiable, thus incidence could be reduced by targeted interventions.

## Background

Birth size is an essential parameter to consider in the clinical evaluation of newborns. Either weight or length at birth represent the expression of growth in utero as a result of maternal, placental, and fetal factors, and have been historically used to identify newborns at potential risk of complications early in life [1]. In addition, there is growing evidence that size at birth is associated with cardio-metabolic outcomes later in life [2]. With respect to the Gaussian distribution of birth size, two main groups have been recognised at increased risk of adverse perinatal outcomes and future cardio-metabolic alterations: infants born small- (SGA) and large-for-gestational-age (LGA) [1, 2].

There is considerable variation in the prevalence of infants born SGA (4.6–15.3 % across Europe) [3] and LGA (5–20 % in developed countries) [4]. These ranges appear even wider in developing countries. It has been estimated that 27 % of all live births were SGA in low- and middle-income countries (more than 32 million infants) in 2010 [5], with the prevalence of SGA babies ranging from 5.3 % in China to 41.5 % in Pakistan [6]. There is also large variation reported on the prevalence of babies with high birthweight (≥4000 g) in developing countries, ranging from 0.5 % in India to 14.9 % in Algeria [7]. The variability in SGA and LGA rates reflects not only socio-environmental factors and population differences, but also the application of different standards across studies [7, 8].

Size at birth has been reported to vary over generations [9–11]. During the second half of the 19th century, there were documented downward trends in birthweight (by approximately 430 g) in Montreal, Canada [9], and in Norway [10], possibly reflecting unfavourable environmental conditions. Between 1900 and 1940 there was an observed rise in birthweight (~150 g) in Norway, followed by another reduction from 1940 to 1984 [10]. However, over the last three to four decades, there has been an increase in birthweight reported in Europe [11] and Australia [12]. For example, among term infants in Canada, the proportion of babies born SGA decreased from 11.1 to 7.2 %, while LGA births increased from 8.0 to 11.5 % over an 18-year period (1978–1996) [13]. Conversely, a decline in birthweight has recently been observed in some countries, such as France [14] and USA [15, 16]. The reasons underpinning the observed variations over time are unclear, but temporal increases in birthweight are said to mirror the increasing maternal adiposity and nutritional excess in utero [17], as maternal factors directly affect fetal growth [8, 18].

As a result, we aimed to assess the trends in SGA and LGA births over a 20-year period (1993–2013) in Chieti (Italy). In addition, we investigated the maternal risk factors associated with SGA and LGA births.

## Methods

Ethics approval for this study was not required, since i) it was an audit; ii) it was confined to anonymised and unidentifiable data that are routinely collected at the S.S. Annunziata Hospital (Chieti); and iii) study findings would not affect patient care.

The province of Chieti (Abruzzo region, east coast of central Italy) has a population of 397,395 (2012 census), mostly of Caucasian origin. Data were retrospectively collected for every 5^th^ year on all infants born in the Department of Obstetrics and Gynecology, S.S. Annunziata Hospital, between 1^st^ January 1993 and 31^st^ December 2013 (specifically for 1993, 1998, 2003, 2008, and 2013). This is the largest maternity hospital in the province of Chieti, covering approximately half of all births.

A retrospective review of obstetric and delivery records was performed, including information on sex, gestational age, anthropometry (birthweight and length), and birth order. Note that when assessing birth order, miscarriages at less than 20 weeks of gestation were disregarded [19]. Gestational age (recorded in completed weeks plus days) was obtained from the interval between the date of last menstrual period and the date of birth, and ascertained on ultrasound assessment within the first trimester. Birth anthropometry was obtained by trained personnel: birthweight to the nearest 10 g using electronic infant scales, and crown-heel length using a Harpenden neonatometer. To estimate birthweight and birth length percentiles and their respective standard deviation scores (SDS), an online calculator was used, based on date of birth, gestational age, sex, birth order, and anthropometry (http://www.inescharts.com/). According to the Italian Neonatal Study (INeS) reference charts [20], newborns were categorized into three groups: appropriate-for-gestational-age (AGA), defined on a birthweight and length between 10^th^ and 90^th^ percentile (−1.28 to 1.28 SDS); SGA, defined on a birthweight and/or length <10^th^ percentile (<−1.28 SD); and LGA, defined on a birthweight and/or length >90^th^ percentile (>1.28 SD). Classification of newborns from multiple pregnancies as SGA or LGA was based on specific percentile curves [21]. Unless otherwise stated, in this study SGA and LGA groups have been defined by weight.

Maternal height, pre-pregnancy BMI, conception (natural or by artificial reproductive technology), birth (multiple vs singleton), and delivery (vaginal or cesarean section) were recorded. Maternal factors, such as previous miscarriage, parity, chronic illnesses, gestational diabetes, hypertension (including pre-eclampsia) [22], and use of recreational drugs, tobacco, or alcohol during pregnancy were also recorded.

The factors associated with SGA or LGA birth were identified using stepwise linear regression analysis with significance levels of 0.4 and 0.2 required for a variable to enter and stay in the model, respectively (SAS v.9.4, SAS Institute Inc. Cary NC, USA). The various parameters included at model entry were: birth year, gestational diabetes, maternal hypertension, maternal ethnicity, maternal age, smoking during pregnancy, alcohol consumption during pregnancy, previous miscarriage, conception (natural vs artificial reproductive technology), birth (multiple vs singleton), sex, and birth order. Subsequent models were also run on the subgroup with maternal anthropometric data, with the addition of maternal height and BMI into the models. Odds ratios (OR) are provided with 95 % confidence intervals in brackets. The agreement in SGA or LGA classifications according to birthweight and birth length was evaluated using the Cohen’s kappa statistic. All statistical tests were two-tailed and significance was maintained at the 5 % level.

## Results

There were a pooled total of 5896 live births recorded across the 5 specific years covered by this study, mostly of Caucasian ethnicity (97.5 %). Detailed pregnancy information and birthweight data were available for 5759 live births (97.7 %).

**Fig. 1:**
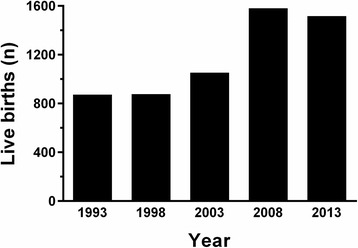
Number of live births at the S.S. Annunziata Hospital in Chieti in 1993, 1998, 2003, 2008, and 2013

**Fig. 2:**
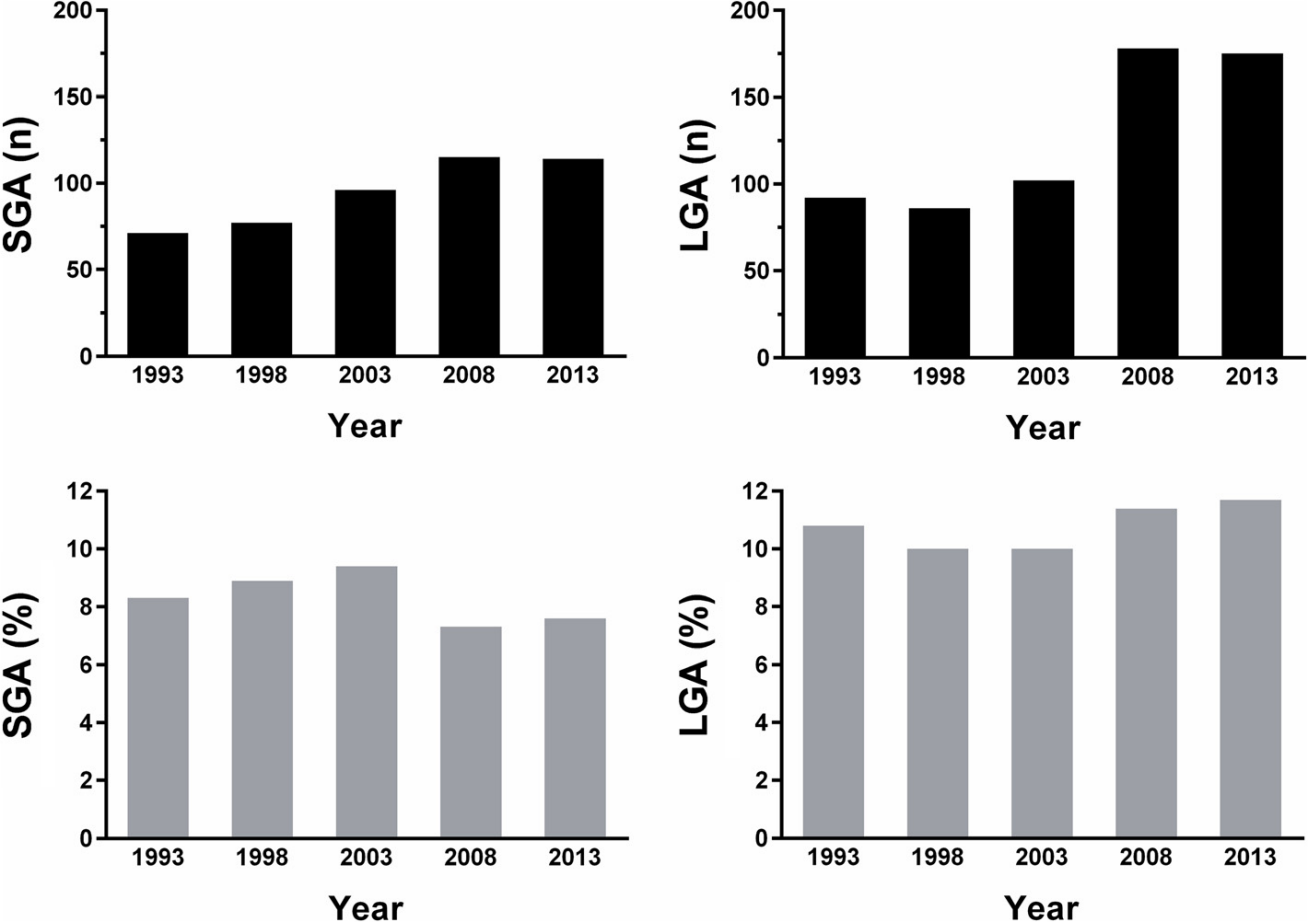
Number (*black*) and incidence (*gray*) of infants born small-for-gestational-age (SGA) and large-for-gestational-age (LGA) at the S.S. Annunziata Hospital in Chieti in 1993, 1998, 2003, 2008, and 2013. Newborns were classified according to birthweight

Pregnancy outcomes for the whole cohort are reported in Table 1. Number of live births increased from 872 in 1993 to 1516 in 2013 (+73.9 %; Fig. 1). There was a corresponding increase in the number of babies born SGA (71/year to 114/year; +60.6 %; Fig. 2) and LGA (92/year to 175/year; +90.2 %; Fig. 2) from 1993 to 2013. However, there were no marked changes in the incidence of SGA or LGA births: 8.3 and 10.8 % in 1993 versus 7.6 and 11.7 % in 2013, respectively (Fig. 2).

**Table 1:** Maternal pathologies and pregnancy outcomes observed amongst births recorded in the S.S. Annunziata Hospital in Chieti in 1993, 1998, 2003, 2008, and 2013. Gestational age and birthweight data are means ± standard deviations; other data are *n* (% out of total cohort)

		1993	1998	2003	2008	2013	Overall
*n*		872	876	1052	1580	1516	5896
Maternal pathologies	Type 1 diabetes	0	0	2 (0.2 %)	2 (0.1 %)	6 (0.4 %)	10 (0.2 %)
	Type 2 diabetes	0	0	1 (0.1 %)	0	1 (0.1 %)	2 (0.03 %)
	Gestational diabetes	7 (0.8 %)	26 (3.0 %)	40 (3.8 %)	57 (3.6 %)	95 (6.3 %)	225 (3.8 %)
	Hypertension	28 (3.2 %)	54 (6.2 %)	59 (5.6 %)	77 (4.9 %)	46 (3.0 %)	264 (4.5 %)
Pregnancy outcomes	Gestational age (weeks)	38.7 ± 2.0	38.7 ± 1.9	38.3 ± 2.6	38.3 ± 2.6	38.6 ± 2.3	38.5 ± 2.3
	Birthweight (kg)	3.24 ± 0.54	3.20 ± 0.54	3.16 ± 0.59	3.12 ± 0.65	3.20 ± 0.58	3.18 ± 0.59
	Preterm	71 (8.2 %)	71 (8.2 %)	142 (13.5 %)	263 (16.7 %)	176 (11.6 %)	723 (12.3 %)
	Multiple birth	31 (3.6 %)	16 (1.8 %)	49 (4.7 %)	114 (7.2 %)	83 (5.5 %)	293 (5.0 %)
	Sex (male)	429 (49.3 %)	463 (52.9 %)	550 (52.4 %)	786 (50.0 %)	779 (51.7 %)	3007 (51.0 %)

### Risk factors

The odds of being born SGA were greater in the offspring of mothers who smoked tobacco during pregnancy (OR 1.49 (1.05–2.14); *p* = 0.028), were hypertensive (OR 2.93 (2.11–4.06); *p* < 0.0001), or had a previous miscarriage (OR 1.35 (1.08–1.68); *p* = 0.008).

Conversely, the odds of being born LGA were greater in males (OR 1.21 (1.02–1.43); *p* = 0.028), and in those born to mothers with gestational diabetes (OR 1.80 (1.25–2.57); *p* = 0.001) or who did not smoke during pregnancy (OR 1.61 (1.04–2.48); *p* = 0.032).

### Maternal anthropometry

Maternal anthropometric data were available for a subset of 3071 live births (52.1 %), and these data were re-analysed incorporating maternal pre-pregnancy BMI and maternal height into stepwise regression analysis. For every 1 kg/m^2^ increase in BMI there were higher odds of having a LGA baby (OR 1.10 (1.07–1.13); *p* < 0.0001) but lower odds of having a SGA baby (OR 0.94 (0.91–0.98); *p* = 0.002). The odds for every 1 cm increase in height followed an identical pattern: being for LGA 1.06 (1.04–1.09; *p* < 0.0001) and for SGA 0.94 (0.92–0.96; *p* < 0.0001).

### Birthweight versus birth length

Data on birth length were available for 3792 infants (64.3 %), as this parameter was not routinely recorded until the last decade. As a result, birth length data were available for 10.9 % of births in 1993, 6.2 % in 1998, 70.2 % in 2003, 94.5 % in 2008, and 93.1 % in 2013. Maternal pathologies and pregnancy outcomes observed amongst these births are reported in Table 2. In this subgroup, 4.5 % were SGA according to weight, 3.4 % according to length, and 3.3 % according to both (Table 3). For LGA, the respective figures were 5.5, 5.9, and 5.6 % (Table 4). There was only moderate agreement between the classification of infants according to birthweight or birth length into SGA (kappa = 0.41; Table 3) and LGA (kappa = 0.44; Table 4).

**Table 2:** Maternal pathologies and pregnancy outcomes observed amongst births with complete infant anthropometry, which were recorded in the S.S. Annunziata Hospital in Chieti in 1993, 1998, 2003, 2008, and 2013. Gestational age and birthweight data are means ± standard deviations; other data are *n* (% out of total cohort)

		1993	1998	2003	2008	2013	Overall
*n*		95	54	739	1493	1411	3792
Maternal pathologies	Type 1 diabetes	0	0	2 (0.3 %)	2 (0.1 %)	6 (0.4 %)	10 (0.3 %)
	Type 2 diabetes	0	0	0	0	1 (0.1 %)	1 (0.03 %)
	Gestational diabetes	0	1 (1.9 %)	32 (4.3 %)	53 (3.6 %)	91 (6.5 %)	177 (4.7 %)
	Hypertension	4 (4.2 %)	2 (3.7 %)	45 (6.1 %)	69 (4.6 %)	39 (2.8 %)	159 (4.2 %)
Pregnancy outcomes	Gestational age (weeks)	39.2 ± 1.0	38.9 ± 1.3	38.6 ± 1.9	38.5 ± 2.0	38.7 ± 2.1	38.6 ± 2.0
	Birthweight (kg)	3.33 ± 0.42	3.32 ± 0.48	3.19 ± 0.52	3.18 ± 0.55	3.21 ± 0.54	3.20 ± 0.54
	Preterm	2 (2.1 %)	3 (5.7 %)	81 (11.0 %)	216 (14.5 %)	158 (11.2 %)	460 (12.1 %)
	Multiple birth	2 (2.1 %)	2 (3.7 %)	20 (2.7 %)	89 (6.0 %)	77 (5.5 %)	190 (5.0 %)
	Sex (male)	48 (50.5 %)	31 (57.4 %)	386 (52.3 %)	744 (49.9 %)	722 (51.2 %)	1931 (51.0 %)

**Table 3:** Agreement in the classification of babies born small-for-gestational-age (SGA) according to birthweight or birth length. Data are *n* (% out of total cohort)

		Birth length		
		SGA	Not SGA	Total
Birthweight	SGA	126 (3.3 %)	172 (4.5 %)	298 (7.9 %)
	Not SGA	129 (3.4 %)	3365 (88.7 %)	3494 (92.1 %)
	Total	255 (6.7 %)	3537 (93.3 %)	3792

**Table 4:** Agreement in the classification of babies born large-for-gestational-age (LGA) according to birthweight or birth length. Data are *n* (% out of total cohort)

		Birth length		
		LGA	Not LGA	Total
Birthweight	LGA	214 (5.6 %)	207 (5.5 %)	421 (11.1 %)
	Not LGA	223 (5.9 %)	3148 (83.0 %)	3371 (88.9 %)
	Total	437 (11.5 %)	3355 (88.5 %)	3792

## Discussion

This study shows that there was an increase in the number of SGA and LGA newborns in Chieti over a 20-year period, but this was a result of increased birth rates. The incidence of SGA and LGA births varied over time, and there were no marked changes observed. Maternal factors associated with increased odds of SGA babies included maternal hypertension, smoking in pregnancy and previous miscarriage, while greater pre-pregnancy BMI and gestational diabetes were risk factors for LGA births.

In a recent prospective cohort data of 75,296 newborns from 12 European countries (1983–2006), the prevalence of SGA births varied from 4.6 % in Finland to 15.3 % in Portugal, with a rate of 9.1 % reported in  Italy (Lazio) [3]. Time trends in SGA births are not consistent across studies. Morisalki et al. found that the proportion of infants born SGA increased from 7.5 to 8.2 % from 2000 to 2008 in Utah and Southeast Idaho (USA) [15]. However, other studies reported a stable or reduced SGA rate over time [13, 23, 24]. In Australia (New South Wales), the rate of SGA births remained stable over a 10-year period (1994–2004) [24]. In contrast, the prevalence of infants born SGA decreased from 15.4 % in 1981–1986 to 8.1 % in 2002–2007 in Québec, Canada [23]. The pattern for SGA births in Chieti over the study period varied over time, but the data indicated a slight increase in the first 10 years (8.3 % vs 9.4 %), with a subsequent decrease in the last decade (back down to 7.6 % in 2013).

In regards to LGA births, there has been a progressive increase in rates reported in several countries over the last decades [12, 13]. Hadfield et al. reported that the proportion of babies born LGA increased from 9.2 to 10.8 % for boys and from 9.1 to 11 % for girls in Australia (New South Wales), from 1990 to 2005 [25]. The increase in LGA births has been observed also in Denmark [11]. In our study, the incidence of LGA births varied but showed the opposite pattern of SGA births in Chieti: decreasing from 10.8 % in 1993 to 10.0 % in 2003, then increasing to 11.7 % in 2013. Similarly, in the United States a decrease in birthweight has been detected from 1990 to 2005, when LGA births steadily dropped from 10.3 % in 2000 to 8.9 % in 2005 [26]. A negative trend in birthweight has also been found in France, with a decrease in the percentage of LGA births from 11 % in 1998 to 9.9 % in 2003 [14]. It has been speculated that this decline may reflect changes in obstetric practices (e.g., decreased gestational length) to prevent perinatal complications in macrosomic fetuses [16].

There are a number of maternal factor known to be associated with an increased risk of delivering a SGA or LGA infant [8, 18]. Consistent with other studies [27, 28], main risk factors for SGA birth in Chieti were maternal hypertension (including pre-eclampsia) and tobacco smoking during pregnancy. Both factors are able to exert damaging effects on the fetus, inducing an abnormal placental development, a decrease in the villous surface exchange and perfusion, with a reduction in fetal nutrition [27, 28]. Women with pre-eclampsia show a near two-fold increase in the rate of intrauterine growth restriction compared to healthy women, with more severe impact on fetal growth when pre-eclampsia develops early in gestation [27]. An increased risk of severe neonatal morbidity and mortality has been also observed in pregnancies complicated by either chronic or gestational hypertension [29, 30]. Even pre-hypertension (systolic blood pressure of 120–139 mmHg or diastolic blood pressure of 80–89 mmHg) [31] in late pregnancy has been associated with an increased risk of having a SGA baby (OR 1.69), with a 2 % increase in risk per each mmHg surge in diastolic blood pressure from early to late pregnancy [32]. Cigarette smoking in pregnancy has been recognized as the major environmental risk factor for SGA babies in Western countries [28]. Of note McCowan et al. found that the rate of SGA births among mothers who had stopped smoking before 15 weeks of gestation was similar to non-smokers and lower than among smokers [33].

A previous miscarriage represented an additional risk factor for SGA birth in Chieti. A spontaneous miscarriage, which occurs in approximately 15 % of clinically recognized pregnancies [34], has been associated with adverse outcomes in a subsequent pregnancy, such as low birthweight and preterm birth [35]. A higher risk of low birthweight (OR 1.6) has been reported in infants born after previous miscarriages (before 24 weeks of gestation) compared to women with one previous successful pregnancy [36]. The cause of low birthweight in neonates born after a previous miscarriage may be related to maternal pathology. Indeed, uterine anomalies, endocrine abnormalities, autoimmune disorders (e.g., antiphospholipid syndrome), and genetic defects are well-established aetiologies for pregnancy loss [37]. Of note, women who have miscarried early in their first pregnancy may virtually act as primipara in the following pregnancy in terms of maternal complications and neonatal outcomes [36].

In accordance with existing evidence [18, 38], we observed that maternal pre-pregnancy BMI and gestational diabetes were associated with increased odds of LGA births. Alberico et al. found that maternal obesity, excessive gestational weight gain, and diabetes were independent predictors of high birthweight [18]. These conditions represent two key components of an ‘obesity cycle’ [17], underpinned by increased nutrient supply to the fetus [39]. Not surprisingly, among healthy women of normal weight in Florida, LGA prevalence has been found to be 5.7 %, increasing to 12.6 and 17.3 % among women who were overweight/obese or had gestational diabetes, respectively [38]. Therefore, temporal trends in maternal factors may explain concurrent trends in LGA births, given the worldwide increase in rates of obesity and gestational diabetes in recent decades [18].

Infants can be defined as SGA or LGA based on birthweight, birth length, or both [8]. In our cohort we observed only moderate agreement between the classification of SGA and LGA infants according to either birthweight or birth length. We speculate that the use of both scales concurrently may provide useful health information, not only in the perinatal period but also for assessing endocrine and metabolic risks later in life. In particular, infants classified as SGA according to both weight and length are likely to have been exposed to early in utero stress, while infants classified as SGA according to weight but not length are likely to result from adverse intrauterine conditions occurring later in pregnancy. Thus, these two different groups of SGA may experience different growth patterns and distinct metabolic outcomes [8]. For instance, infants defined as SGA for length have an increased risk of short stature than those defined as SGA for weight [40], possibly requiring closer endocrine monitoring. Conversely, those babies who were LGA based on weight but not on length likely represent those with the greatest adiposity [41], and a systematic review showed that infants who are larger based on weight are at greater risk of later obesity [42]. Ahlsson et al. found that adult women born LGA based on both weight and length had a lower risk of obesity than those born LGA according to weight but not length, with the latter showing a 40 % increased risk of being overweight than women born AGA [43]. Thus, the discrepancy or agreement between the two scales could identify contrasting SGA and LGA phenotypes and be good indicators of long-term health outcomes. It would be of interest to further explore this issue in future studies.

The main limitation of our study was the lack of complete maternal and newborn data on the whole cohort. In particular, the reduced number of infants with maternal anthropometric and/or birth length data could possibly limit the applicability of specific findings across the whole population. In addition, we only provide annual data collected every 5^th^ year, so that it is not possible to ascertain what the incidence of SGA and LGA births was in the intervening years. Nonetheless, our study covers comprehensive pregnancy information and birthweight data.

## Conclusions

In summary, the incidence of SGA and LGA births has not markedly changed in Chieti over the last two decades, even though numbers of babies born small and large have increased due to a growing population. However, most of the maternal factors observed to be associated with increased odds of SGA and LGA births are modifiable. Thus, it is important that interventions target women both before and during pregnancy to reduce SGA and LGA incidence, and consequently minimize potential short- and long-term adverse effects on offspring health.
